# Trichoblastic Carcinoma in the Glabella Treated With Mohs Micrographic Surgery

**DOI:** 10.7759/cureus.63060

**Published:** 2024-06-24

**Authors:** Lily Park, David Crasto, Shelby L Kubicki, Daniel Rivlin

**Affiliations:** 1 Department of Dermatology, Larkin Community Hospital, Nova Southeastern University, South Miami, USA; 2 Department of Mohs Micrographic Surgery, Larkin Community Hospital, Nova Southeastern University, South Miami, USA

**Keywords:** cosmetic surgery, dermatologic surgery, non-melanoma skin cancers, facial cosmetic surgery, mohs micrographic surgery, dermatologic oncology, cosmetic dermatologic surgery, dermatology and dermatologic surgery, malignant trichoblastoma, trichoblastic carcinoma

## Abstract

Trichoblastic carcinoma (TBC) is a rare adnexal neoplasm of follicular germ cell differentiation with the potential for local invasion and metastasis. Histologic features of trichoblastic carcinoma have significant overlap with trichoblastoma and basal cell carcinoma (BCC), making diagnosis difficult in some cases. Treatment strategies are not well defined and include surgical excision for localized tumors and systemic therapies for metastatic disease. We present a case of trichoblastic carcinoma clinically resembling a benign cyst that was ultimately treated with Mohs micrographic surgery (MMS).

## Introduction

Trichoblastic carcinoma (TBC), or malignant trichoblastoma, is a rare malignant adnexal neoplasm. TBC is conventionally thought to develop within pre-existing trichoblastoma [1] but can also occur within trichoepithelioma in conjunction with genodermatoses such as multiple familial trichoepitheliomas or Brooke-Spiegler syndrome [2-4]. In contrast to prior views, a recent retrospective review reported that most cases (87%) seemed to have occurred spontaneously, though the authors noted that pre-existing benign lesions might have gone undetected before the diagnosis of TBC [5]. The pathophysiology of TBC is not well understood. Abnormal activation of phosphatidylinositol 3-kinase (PI3-AKT) signaling pathways or a p53 mutation resulting in tumor suppressor function loss may be associated with the development of TBC [6,7]. Cyclin-dependent kinase inhibitor 2A (CDKN2A) gene mutations have also been observed in some cases [8]. TBC has not been studied extensively due to its rarity. As a result, diagnostic characteristics, management guidelines, prognosis, and clinical outcomes remain poorly defined [5]. In this report, we present a case of TBC that clinically resembled a benign cyst that was ultimately treated with Mohs micrographic surgery (MMS).

## Case presentation

**Figure 1:**
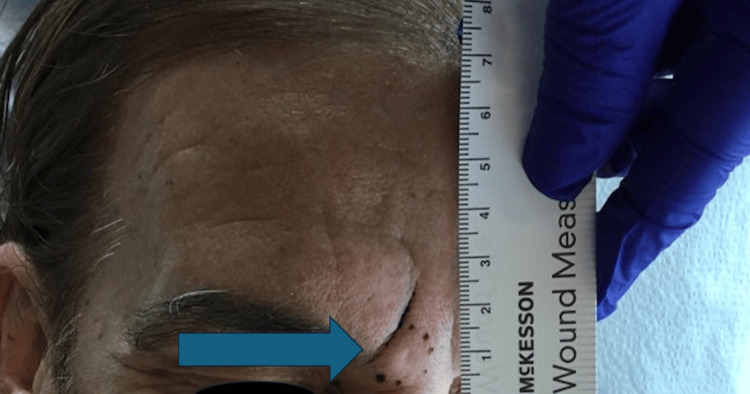
A 1 cm × 1 cm subcutaneous nodule with a central follicular opening in the glabella

A 65-year-old white man with no past medical history other than a history of anal cancer in remission presented with a firm, mobile, 1 cm × 1 cm subcutaneous nodule with a prominent follicular opening growing slowly for one year in the glabellar region of the forehead (Figure 1). He had no history of radiation to the affected area and was not on any immunosuppression. The lesion was asymptomatic, and no epidermal change was observed. The lesion was clinically diagnosed as a cyst, and the patient was scheduled for excision.

**Figure 2:**
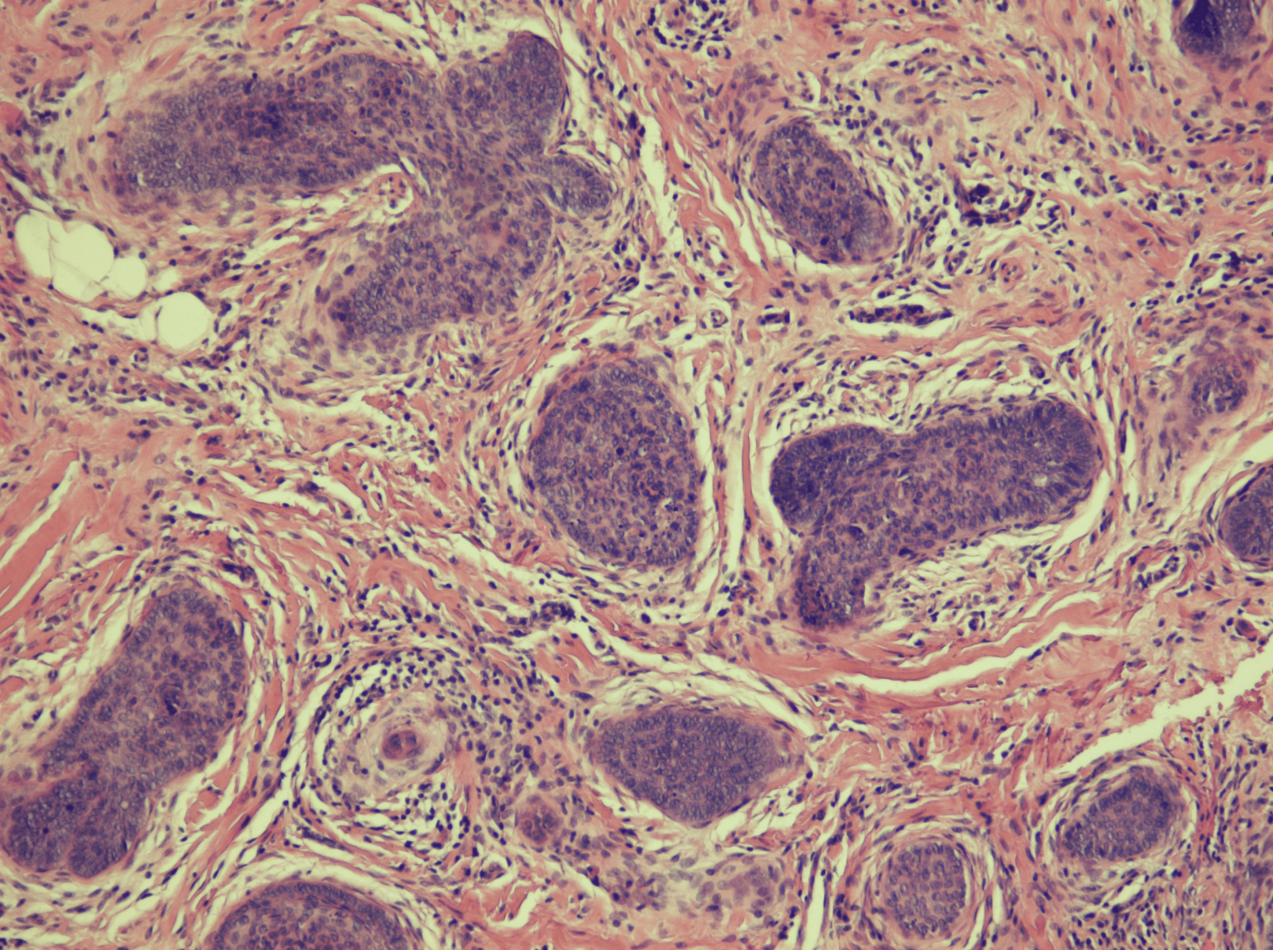
Histopathologic images of trichoblastic carcinoma

Preoperatively, the site was injected with 18 units of abobotulinumtoxin A to reduce movement around the nodule and minimize the scar after the excision. An excision was performed a week later. Histologic findings demonstrated aggregations of palisaded basaloid cells surrounded by concentrically arranged fibrocytes and a collagenous stroma without significant epithelial-stromal clefting. Trichoblastoma was initially considered, but due to the unusual presentation, a second opinion was sought. The final diagnosis of TBC was made based on the infiltrative pattern extending nearly to the subcutis and the overall poorly circumscribed appearance. Notably, the aggregates of basaloid cells were not collectively contained within a smooth-bordered stroma, but each carried a smaller cuff of stroma (Figure 2).

Since the patient did not present with occult lymphadenopathy, systemic symptoms, or high-risk features such as perineural invasion, lesion size greater than 2 cm, or immunosuppression, no sentinel lymph node biopsy or imaging was performed to assess for distant metastasis. The patient underwent MMS to ensure the complete removal of the tumor. No residual TBC was observed on frozen sections, and a complex linear closure was performed. He will be followed up in six months for surveillance and a full skin check.

## Discussion

TBC is a rare follicular malignant neoplasm with the potential for aggressive behavior. Initially referred to as “malignant trichoblastoma," the nomenclature “trichoblastic carcinoma” was introduced in 2000 [9]. Common locations for TBC include the face (48%), followed by the trunk (18%), scalp (14%), and extremities (13%), according to a recent retrospective literature review of 93 patients [5].

TBCs are categorized into low-grade tumors that remain localized and high-grade tumors, which have a higher risk of recurrence and metastasis. Overall, the risk of metastasis ranges from 8.6 to 11% [5,6,10]. Low-grade TBC can be misdiagnosed as basal cell carcinoma (BCC) with follicular differentiation due to similar histologic features. Due to their resemblance, Ackerman et al. initially proposed classifying these tumors together; however, the consensus is to refer to them separately given the more aggressive clinical behavior of TBC [11]. TBC has been linked to mutations in the CYLD gene and is observed in patients with multiple familial trichoepithelioma (MFT) and Brooke-Spiegler syndrome, which is an autosomal dominant disease characterized by the growth of trichoepitheliomas, spiradenomas, cylindromas, or their combination [12].

Histologically, TBC can be distinguished from BCC by the follicular pattern of cells as well as the absence of several BCC-specific features, including retraction artifacts, stromal edema and lymphocytes, connection to the epidermis, and ulceration [13]. TBC exhibits the presence of a hypercellular, fibrous, non-myxoid stroma and is frequently accompanied by an adjacent benign follicular neoplasm with a clear transition zone. Numerous mitoses and extensive necrosis can be seen, especially in high-grade tumors [1,6,13,14]. Immunohistochemical staining, such as PHLDA1 (pleckstrin homology-like domain, family A, member 1), a marker of hair follicle stem cells, can be of value in distinguishing TBC from BCC in many cases [15-17]. TBC presenting as a subcutaneous nodule, as in our case, may clinically resemble a cyst or lipoma. In these patients, ultrasound may be a useful diagnostic modality [18].

Currently, there is no established consensus for treating TBC. Surgical removal with wide local excision to the deep fascia with a lateral margin of 10 mm or greater has been a conventional treatment for TBC [16]. MMS has emerged as another treatment to increase histologic margin assessment and maximize tissue sparing in cosmetically sensitive areas such as the head and neck, as in our case [19]. While the radiosensitivity of adnexal carcinomas remains uncertain, radiotherapy may be considered a standalone or combination treatment modality for locally advanced or inoperable cases.

Systemic therapy is also utilized for advanced diseases. Sunitinib, an oral tyrosine kinase inhibitor, achieved a partial response in one patient with metastatic TBC [20]. Additionally, there is a recent report of an association between a low-grade malignant variant of trichoblastoma and sonic hedgehog pathway mutations [21]. Vismodegib, an FDA-approved sonic hedgehog pathway inhibitor indicated for locally advanced or metastatic BCC, has exhibited promising results in the treatment of advanced TBC with better tolerability than sunitinib [17,20,22]. A recent phase II study with nivolumab, a programmed cell death protein 1 (PD-1) inhibitor, also demonstrated promising results with an 80% one-year survival rate and 54.5% progression-free survival at six months in 11 patients with advanced TBC [23].

## Conclusions

TBC is a rare follicular malignancy with the potential for local invasion and metastasis. The ability to differentiate between TBC, trichoblastoma, and BCC is imperative, given differences in metastatic potential. Additional research is crucial for developing clear guidelines on the diagnosis, prognosis, and treatment of this rare neoplasm.
